# Methods of competing risks analysis of end-stage renal disease and mortality among people with diabetes

**DOI:** 10.1186/1471-2288-10-97

**Published:** 2010-10-21

**Authors:** Hyun J Lim, Xu Zhang, Roland Dyck, Nathaniel Osgood

**Affiliations:** 1 Department of Community Health & Epidemiology College of Medicine, University of Saskatchewan 107 Wiggins Road Saskatoon, SK S7N 5E5, Canada; 2 Department of Mathematics &Statistics Georgia State University 750 COE, 7th floor, 30 Pryor Street Atlanta, Georgia 30303, USA; 3 Department of Medicine, University of Saskatchewan 103 Hospital Drive Saskatoon, SK S7J 5B6, Canada; 4 Department of Computer Science University of Saskatchewan 110 Science Place Saskatoon, SK S7N 5C9, Canada

## Abstract

**Background:**

When a patient experiences an event other than the one of interest in the study, usually the probability of experiencing the event of interest is altered. By contrast, disease-free survival time analysis by standard methods, such as the Kaplan-Meier method and the standard Cox model, does not distinguish different causes in the presence of competing risks. Alternative approaches use the cumulative incidence estimator by the Cox models on cause-specific and on subdistribution hazards models. We applied cause-specific and subdistribution hazards models to a diabetes dataset with two competing risks (end-stage renal disease (ESRD) or death without ESRD) to measure the relative effects of covariates and cumulative incidence functions.

**Results:**

In this study, the cumulative incidence curve of the risk of ESRD by the cause-specific hazards model was revealed to be higher than the curves generated by the subdistribution hazards model. However, the cumulative incidence curves of risk of death without ESRD based on those three models were very similar.

**Conclusions:**

In analysis of competing risk data, it is important to present both the results of the event of interest and the results of competing risks. We recommend using either the cause-specific hazards model or the subdistribution hazards model for a dominant risk. However, for a minor risk, we do not recommend the subdistribution hazards model and a cause-specific hazards model is more appropriate. Focusing the interpretation on one or a few causes and ignoring the other causes is always associated with a risk of overlooking important features which may influence our interpretation.

## 1. Background

**Figure 1:**
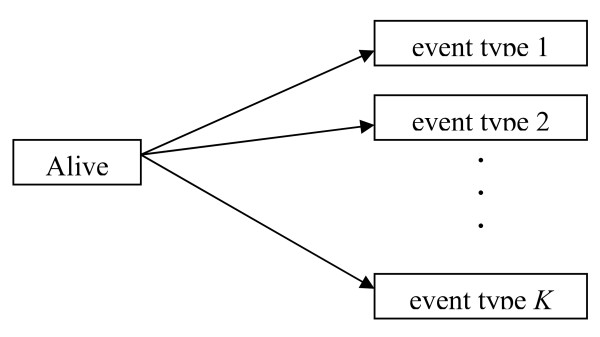
Competing risk models with *K *different event types

In medical research, each person studied can experience one of several different types of events over the follow-up period and survival times are subject to competing risks if the occurrence of one event type prevents other event types from occurring. For example, in a study of bone marrow transplantation, leukemia relapse and death in remission are competing risks [1,2]. Leukemia relapse will not be observed once patients have died. Similarly, in a study of people with diabetes, end-stage renal disease (ESRD) and death compete for the life of the person, and each influence the risk of the other [3,4]. When a person experiences an event other than the one of interest in the study, the probability of experiencing the event of interest is frequently altered. Thus, caution is needed when we estimate survival probability of the event of interest in competing risks analysis [5]. Accordingly, if a person reaches the primary event of interest (e.g, ESRD), the other event (e.g, death without ESRD) is censored. The competing risk model can be described by specifying the cause-specific hazards as visualized as in Figure 1.

With competing risks data, the nonparametric Kaplan-Meier [6] cumulative hazard function, {1 - *S*_*KM*_*(t)*}, has been used in some research. However, studies have demonstrated that {1 - *S*_*KM*_*(t)*} is inappropriate because it overestimates the probability of occurrence of the event of interest [7-12]. The bias is especially great when the hazard of the competing events is large [13]. An alternative method to the inappropriate cumulative hazard function is Cox cause-specific hazard [14] and cumulative incidence functions (CIF), which are the most important approaches to analyse competing risks data [12]. The cause-specific hazard measures the instantaneous failure rate due to one risk at a time. It is routinely estimated by constructing the Cox models on cause-specific hazards and treating time to event from the other competing risks as censored [11,12]. For each risk, the effects of prognostic factors are assessed as constant hazards ratios on the instantaneous failure rate of this risk. The CIF is an important quantity related to one risk in the context of competing risks. The CIF curve provides a better incidence curve associated with one risk than {1 - *S*_*KM*_*(t)*}. It also provides a meaningful interpretation in terms of failure due to one risk regardless of whether competing risks are independent. Comparing the CIF curves is analogous to the log-rank test and is identical to the log-rank test in the absence of competing risks [15]. Gray considered a class of *K*-sample tests for the cumulative incidence based on weighted averages of subdistribution hazard functions [15]. Such tests do not require the independence assumption and does not adjust for other covariates.

In recent years, research methods centered on directly assessing covariate effects on a CIF have been developed [16,17]. One important work is the proportional subdistribution hazards model proposed by Fine and Gray [16]. This approach directly measures the covariate effects on the cumulative failure probability due to one risk, in the presence of other risks. As in any other regression analysis, modeling CIF for competing risks can be used to identify potential prognostic factors for a particular event in the presence of competing risks, or to assess a prognostic factor of interest after adjusting for other potential risk factors in the model.

The primary aim of this paper is to apply regression models on cause-specific hazards and subdistribution hazards to people with diabetes and to examine the estimates obtained by such models. We have identified the competing risk of "ESRD" versus "death without ESRD" in a diabetes population and evaluate the risk factors that are associated with these two outcomes. In the next section, we introduce a description of the diabetes study. In Section 3, models on cause-specific and subdistribution hazards for analysis are reviewed. In Section 4, the models are applied to the diabetes dataset to measure the hazard ratios of covariates and the cumulative incidence function. Section 5 contains a discussion about results and conclusions.

## 2. Study Description

### 2.1. Clinical Background

Diabetes is one of the most common chronic diseases occurring globally. In developed countries, population aging, inactivity, growing prevalence of obesity, and improved management of chronic complications have contributed to an epidemic of type 2 diabetes (T2DM) [4,18-23]. T2DM is particularly common among African Americans, Latinos, Native Americans, and Asian Americans/Pacific Islanders [24]. Increasing rates of T2DM among African-American and Canada's First Nations peoples reflect a parallel epidemic of overweight/obesity caused in large part by disruption of traditional cultures and lifestyles [25-27]. In addition to the above, risk factors for diabetes include obesity, physical inactivity, advanced aging, high blood pressure and/or high cholesterol, and family history of diabetes [24,26,28]. It has been reported that T2DM contributes to and is associated with increased mortality in end-stage renal disease (ESRD) populations [18,28,30]. Diabetic nephropathy affects about 10-20% of people with diabetes [31,32] and is a leading cause of ESRD [21-23,31]. Furthermore, studies have shown that individuals with both diabetes and ESRD have higher morbidity and mortality rates than individuals with only one of these conditions [4,33,34]. Finally, it has also been reported that women with ESRD have worse outcomes compared to men [4,28,29].

### 2.2 Study Population

We conducted a population study of diabetes, utilizing data drawn from the Saskatchewan Ministry of Health administrative databases. Descriptions of the overall study design and profile have been published elsewhere [27]. Briefly, Saskatchewan is a mid-western province of Canada with a population of approximately one million people through the years of study. Approximately 99% of the provincial population are beneficiaries of a universal health care system and recorded in the Ministry of Health's insurance registry. For this sub-study, 8274 First Nations people age 20 years or older with a diabetes incident year between 1980 and 2005 were identified as registered Indians in the databases.

In this study we excluded diabetes records related to a gestational record to ensure that gestational diabetes cases were not counted as diabetes cases. The ESRD case definition was based on physician fee-for-service codes for chronic dialysis and renal transplantation. To qualify as a chronic dialysis patient in the study, a person was required to have received dialysis for at least 90 days, and to have received that treatment without any break of 21 days or greater. We excluded 20 patients who were classified as having reached ESRD prior to diabetes diagnosis. For all patients in the study, we obtained the following information: birth year, sex, and diabetes incident year. Where applicable, the ESRD incident year, year of death, and any period of health care coverage loss were also provided. A competing risk model was used to analyze the risk of two event types - ESRD or death without ESRD. Censoring time was set at December 31, 2005. In this study we explored and determined the effect of diabetes on ESRD and death when demographic characteristics were taken into account in the competing risks analysis.

## 3. Models

### 3.1. Standard single event time model

Let *T *be a random variable representing survival time that has a density function, *f(t)*, and the distribution function, *F(t)*. The survival function at time *t*, *S(t)*, is defined to be the probability that the survival time is greater than *t*, where *S(t) = P(T > t) = *1 - *F*(t). The survival function, therefore, represents the probability that an individual survives from the time origin (for example, time of the study enrollment or disease diagnosis) to sometime beyond *t*. The hazards function or hazard rate, *h(t)*, is the probability that an individual dies at time *t*, conditional on having survived to that time, which is defined as:

$$h(t)=\underset{\Delta t\to 0}{\lim}\;\left\{\frac{P\left(t\leq T<t+\Delta t|T\geq t\right)}{\Delta t}\right\}.$$

The hazard function, therefore, represents the instantaneous death rate for an individual surviving up to time *t *and provides a full characterization of the distribution of *T*. [35].

The main concern with this approach is how to study the impact of important covariates on the distribution of *T*. To do this, we assume the variation in the distribution of event and censoring times can be characterized by a vector of observed explanatory covariates, ***z***, which can be either time-invariant or time-dependent covariates. Under the Cox proportional hazards model, the hazard function for the event time *T *associated with the covariates ***z ***is defined as:

$$h(t)=h_{0}(t)\;e^{\beta^{'}\;Z}.$$

Here, the function *h*_*0*_*(t) *is an unspecified baseline hazard function and gives the shape of the hazard function. If all explanatory covariates are zero, the hazard function will be the baseline hazard *h*_*0*_*(t)*. If two individuals have identical values of the measured covariates, they will have identical hazard functions. The cumulative hazard function given ***z ***is defined by $\text{Λ}(t;z)\;={\text{Λ}}_{0}(t)\;e^{\beta^{'}\;Z}$, where Λ_0_(*t*) is the cumulative baseline hazard and ${\text{Λ}}_{0}(t)=\int_{0}^{t}h_{0}\text{(u)}du$. The survival function is then obtained from the cumulative hazard function such that *S(t) *= *exp*{- *Λ(t; ****z****) *}.

### 3.2. Models on cause-specific hazards

Competing risks in survival analysis refer to a situation where subjects under investigation are exposed to more than one possible type of events. Thus, each subject is associated with a pair (*T*, *D*) where *T *is the time-to-event (event time or failure time) and *D *is the type of the event for that subject. Here we assume that the possible causes are numbered from 1, ..., *K*. The cause-specific hazard function in the competing risks model is the hazard of failing from a given cause *k *in the presence of the competing events

$$h_{k}\left(t\right)=\underset{\Delta t\to 0}{\lim}\left\{\frac{P\left(t\leq T<t+\Delta t,D=k|T\geq t\right)}{\Delta t}\right\}\text{with }D=\text{1},...,K.$$

With covariates, the regression model on cause-specific hazards is *h*_*k*_*(t; ****z****) *= *h*_*0k*_*(t) e ^β Z^*.

The total hazard, *h(t;****z***), equals the value of its corresponding hazards function summed up to time *t*. It is then

$$h(t;z)\;=\sum_{k=1}^{K}h_{k}(t).$$

This equation means that the all-cause hazard rate is the sum of *K *hazards.

Define ${\text{Λ}}_{k}(t;z)={\text{Λ}}_{0k}(t)\;e^{\beta^{'}Z}$, where ${\text{Λ}}_{0k}(t)=\int_{0}^{t}h_{0k}\text{(u)}du$ and *S*_*k*_*(t; ****z****) *= *exp*{- *Λ*_*k *_*(t; ****z****)*}. Although we can estimate *S*_*k*_*(t; ****z****) *from the cause-*k *specific cumulative hazard, *exp*{- *Λ*_*k *_*(t; ****z****)*} is not interpretable as the marginal survival function for cause-*k *specific alone [12]. Instead *S*_*k*_*(t; ****z****) *is the survival probability for the *k*^th ^risk if all other risks were hypothetically removed.

With competing risks data, the cumulative incidence curve derived from cause-specific hazard functions provides important event information for a specific cause. The cause-specific cumulative incidence function (CIF) of cause *k *at time *t, I*_*k*_*(t)*, is defined by the probability of failing from cause *k*,

$$\begin{array}{cc} I_{k}(t)=P\left(T\leq t,D=k\right) & k=\text{1},...,K. \end{array}\;$$

Given the covariate value ***z***, the CIF for cause *k *is also defined as

$$I_{k}(t;z)=\;\int_{0}^{t}S(u;\text{z})\;d{\text{Λ}}_{k}(u;\text{z})=\int_{0}^{t}S(u;\text{z})\;h_{k}\text{(u)}du$$

where *S(t; ****z****) *and *Λ*_*k *_*(t; ****z****) *are the adjusted overall survival and cumulative hazard based on certain types of cause-specific hazard regression models [12]. This expression shows that the cumulative incidence of a specific cause *k *is a function of both the probability of not having the event prior to another event first (*S(u)) *up to time *t *and the cause-specific hazard (*h*_*k*_*(u)*) for the event of interest at that time [7,8,12]. Estimation of the CIF can be obtained by using the cause-specific hazard.

Lunn-McNeil [36] extends to only one Cox model on cause-specific hazards rather than separate cause-specific models for each competing risk. Their method is an adaptation of Cox regression requiring event type indicator variables, which corresponds to different event types.The Lunn-McNeil approach stratified by event type gives identical results to those obtained from separate Cox models. The unstratified Lunn-McNeil model is an unstratified Cox proportional model, which can be used when constant hazard ratios between risk types is assumed. The unstratified Lunn-McNeil method assumes that different risk types have proportional baseline hazard functions. By contrast, the stratified method permits distinct baseline hazards for each event type [17]. If the proportionality assumption is not satisfied, then the stratified Lunn-McNeil model should be used.

### 3.3. Model on a subdistribution hazards

Fine and Gray [16] proposed a regression modeling applied directly on a cumulative incidence function for particular use in competing risks analysis. For any event type, this approach focuses on the hazard associated with the CIF, *I*_*k*_*(t; ****z***), which expresses the effect of covariates directly on the CIF. This is done via the subdistribution hazard function h*_k_*(t; ****z***):

$$h{*}_{k}(t;z)\;=\;h{*}_{0k}(t)\;e^{\beta \;Z}.$$

The CIF on the subdistribution is the function such that

$$I_{k}(t;z)\;=\text{1}-\;\text{exp}\{-\int_{0}^{t}h{*}_{k}\text{(u, z) }du\}.$$

Here h*_k_*(t; ****z***) is not the cause-specific hazard. The CIF for cause *k *not only depends on the hazard of cause *k*, but also on the hazards of all other causes. For this approach, the subdistribution hazard is also defined as

$$\begin{array}{c} \;h{*}_{k}(t;z)\;=\underset{\Delta t\to 0}{\lim}\left\{\frac{P\;\left[t\leq T<t+\Delta t,D=k|T\geq t\cup \text{(}T<t\cap D\neq k)\right]}{\Delta t}\right\}\\ =-\frac{d\log\left\{\;1-I_{k}(t;\text{z})\right\}}{dt} \end{array}$$

so that the covariate effect directly relates to the cumulative incidence function [12]. Fine and Gray imposed a proportional hazards assumption on the subdistribution hazards and gave estimators and large sample properties [12]. This method takes into account other events and does not make any assumptions about their independence between the event time and censoring distribution, i.e., the censoring mechanism is independent of disease progression.

Estimation of the covariates coefficients for the models on cause-specific and subdistribution hazards follows the partial likelihood approach used in the standard Cox model. However, the difference between cause-specific and subdistribution hazards lies in the risk set. For the cause-specific hazard, *h*_*k*_*(t;****z***), the risk set decreases at each time point at which there is an event of another cause. For the subdistribution hazard, h*_k_*(t;****z***), a person who has an event from another cause remains in the risk set [16]. In our study, we have applied the Cox models on the cumulative incidences of ESRD and death without ESRD, and have determined the subdistribution hazards ratios.

## 4. Results

Of the 8254 subjects in the study, 3718 (45%) were male and mean age at diabetes diagnosis was 47.2 (s.d = ± 14) years old. During the study period (median follow-up time = 8.2 years), 1482 (17.9%) subjects died without ESRD and 200 (2.4%) developed ESRD. Of 200 ESRD patients, mean age of ESRD was 56.5 (s.d.= ± 11.2) years old and 110 (55%) died. The events of interest were the times to ESRD, or death without ESRD. The study censoring time was set at December 31, 2005. In contrast to other contributions that focused on describing the study population [27], we used the data below to illustrate the application of the competing risk hazards models and provide comparisons between techniques.

In our study, we applied the Cox models on cause-specific and subdistribution hazards to obtain the cumulative incidences of ESRD and death without ESRD: the hazards ratios are given in Table 1. The results show that the effect sizes from the cause-specific and subdistribution hazards models are quite close for death events but are different for ESRD events. This indicates that the covariates interacted with the two event types. Males were at 51% and 32% higher hazard risks of ESRD compared to females in the cause-specific and subdistribution models, respectively (Table 1). Age at diabetes diagnosis had a significant effect on development of ESRD. Data also showed that age greater than 60 years old was not different compared to age less than 40 years old on the risk of ESRD in the cause-specific model (p-value = 0.144). However, it was significant in the subdistribution model (p-value = 0.0066).

**Table 1:** Estimation of hazard ratio (H.R), 95% confidence interval (C.I), and p-value from the Cox cause-specific and subdistribution hazards models of time from the diabetic diagnosis to ESRD and to death without ESRD

Competing Risk	Model	Covariate	H.R	95% C.I	p-value
ESRD		Male	1.513	1.146 - 1.998	0.004
	Cox				
	cause-specific	Age < 40	-	-	-
	hazards model	40 < Age < 6	1.149	0.849 - 1.554	0.368
		60 < Age	1.405	0.891 - 2.215	0.144
	-----------	-----------	------	-----------	-----
	Cox	Male	1.323	1.006 - 1.742	0.045
	subdistribution				
	hazards model	Age < 40	-	-	-
		40 < Age < 60	0.923	0.685 - 1.243	0.6
		60 < Age	0.53	0.335 - 0.838	0.007

Death without ESRD					
	Cox	Male	1.377	1.243 - 1.525	< 0.0001
	cause-specific				
	hazards model	Age < 40	-	-	-
		40 < Age < 60	2.68	2.267 - 3.158	< 0.0001
		60 < Age	10.23	8.653 - 12.09	< 0.0001
	------------	------------	------	------------	------
	Cox	Male	1.36	1.226 - 1.498	< 0.0001
	subdistribution				
	hazards model	Age < 40	-	-	-
		40 < Age < 60	2.65	2.248 - 3.126	< 0.0001
		60 < Age	9.96	8.453 - 11.74	< 0.0001

Male sex and increasing age were significant predictors for death without ESRD (Table 1). Even when the competing risk of ESRD occurrence was taken into account, males and older age groups had a higher probability of death without ESRD than females and young age groups, respectively. The cause-specific and subdistribution hazards models showed that males faced a 1.37 times higher risk of death without ESRD than females. Risk of death without ESRD also increased with age. People aged 40 to 60 years had 2.65 times higher risk of death compared to those aged younger than 40 years in the subdistribution model (95% C.I: 2.267 - 3.158, p-value < 0.0001). After adjusting for sex, this is interpretable as the risk of death without ESRD for people aged 40 to 60 increasing by 165% compared to people younger than 40.

Table 2 shows the risk of ESRD and death without ESRD when the data were analyzed by the unstratified Lunn-McNeil model under the assumption that the baseline cause specific hazards are proportional. Here the hazard of ESRD and death without ESRD were both increased by 40% for males compared to females. The risk type hazard ratio of 2.44 in the Table 2 indicates that for females younger than 40 years, the hazard of death without ESRD is 2.44 times higher than that of ESRD (95% C.I: 1.788 - 3.328, p-value < 0.0001). As clinically expected, older patients had a higher risk of death, but age did not show an effect on ESRD risk. Note that because the stratified Lunn-McNeil model is identical to the Cox cause-specific model, it is not discussed further.

**Table 2:** Estimation of hazard ratio (H.R), 95% confidence interval (C.I), and p-value from the Lunn-McNeil unstratified models assuming constant ratios between ESRD and death without ESRD

Competing Risk	Covariate	H.R	95% C.I	p-value
ESRD	Male_ESRD_	1.395	1.057 - 1.84	0.0186
	Age_(ESRD) _< 40	-	-	-
	40 < Age_(ESRD) _< 60	1.078	0.797 - 1.457	0.627
	60 < Age_(ESRD)_	1.004	0.641 - 1.574	0.985
	-------------------------	------------	-----------------	----------------
Death without ESRD	Risk type *	2.44	1.788 - 3.328	< 0.0001
	Male_(death)_	1.40	1.264 - 1.55	< 0.0001
	Age_(death) _< 40	-	-	-
	40 < Age_(death) _< 60	2.708	2.291 - 3.202	< 0.0001
	60 < Age_(death)_	10.73	9.078 - 12.68	< 0.0001

* The reference competing risk type is ESRD.

**Figure 2:**
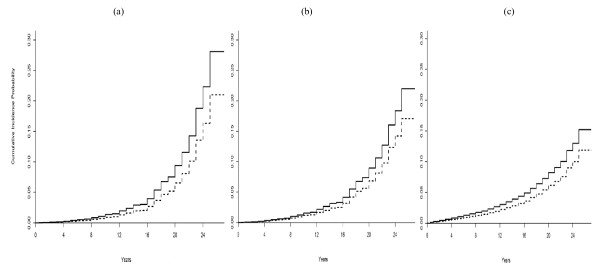
**2a-2c: Estimates of the cumulative incidence curves of risk of ESRD**. Estimates were by sex for subjects younger than 40 years old patient based on (a) the cause-specific hazards model; (b) the subdistribution hazards model; (c) the unstratified Lunn-McNeil model. Dashed line is for males and dotted line is for females.

**Figure 3:**
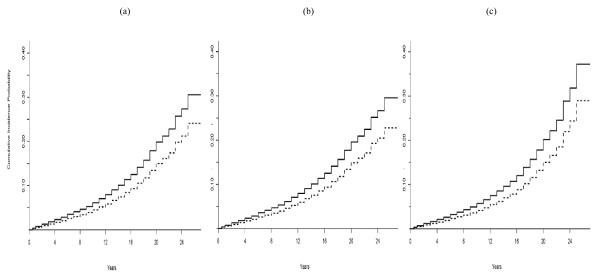
**3a-3c: Estimates of the cumulative incidence curves of risk of death without ERSD**. Estimates were by sex for subjects younger than 40 years old patient based on (a) the cause-specific hazards model; (b) the subdistribution hazards model; (c) the unstratified Lunn-McNeil model. Dashed line is for males and dotted line is for females.

Figures 2a-c and Figures 3a-c show the estimates of the CIF curves of risk of ESRD and death without ESRD by sex for subjects younger than 40 based on the Cox cause-specific, subdistribution hazards models, and the unstratified Lunn-McNeil model. Estimates for the cause-specific hazards model provided a slightly higher CIF curve than for the subdistribution hazards model and the unstratified Lunn-McNeil model (Figures 2a-c). The cumulative incidence probability of ESRD approached 6.6% in females and 9.4% in males in the cause-specific model (Figure 2a), 6.8% in females and 8.9% in males in the subdistribution model (Figure 2b), and 6.2% in females and 8.2% in males in the unstratified Lunn-McNeil model (Figure 2c) at 20 year after diabetes diagnosis.

Figures 3a-c show the estimates of the CIF curves of risk of death without ESRD by sex for subjects younger than 40 based on the cause-specific and subdistribution hazards models, and the unstratified Lunn-McNeil model. The estimated CIF curves based on the three models are almost identical for the first 22 years. The cumulative incidence probability of death approached 15% in females and 20% in males at 20 years after diabetes diagnosis.

## 5. Discussion

In this study we used diabetes data to demonstrate analyses for competing risks and showed the differences in estimates obtained by the cause-specific and subdistribution hazards models, and the Lunn-McNeil model. Our analyses showed that the three models yielded different results with regard to the effects of covariates. The CIF of the cause-specific hazards model revealed a higher CIF curve than the subdistribution hazards model: the unstratified Lunn-McNeil model was lower yet. However, the cumulative incidence curves of risk of death without ESRD on those three models were very similar. Our data showed such noticeable phenomenon consistently throughout the other covariate (age, sex) categories.

The Kaplan-Meier survival estimate is not applicable for competing risks analysis. The Cox proportional regression approach requires a proportionality assumption. If the number of competing events is large or some events are rare, the proportionality assumption is often not satisfied. When the proportionality assumption is violated, the effects of covariate on the CIF curve can no longer be expressed by a simple number [13]. Gooley showed how the cumulative incidence curve can also be obtained [37]. The cumulative incidence of a specific event *k *is a function of both the probability of not having the event prior to another event (*S(u)) *up to time *t *and the cause-specific hazard (*h*_*k*_*(u)*) for the event of interest at that time [7,8,12].

Our study showed that the estimates of the covariates coefficients on the cause-specific hazards and on the subdistribution hazards models were different. Latouche et al showed that the effects of covariate on the cause-specific hazard and on the subdistribution hazard were normally different [38]. Their paper addressed the relationship between the Cox cause-specific and subdistribution hazards models using a simulation study. The cumulative incidence of the minor (smaller number of events) risk may be greatly governed by the outcome of the dominant (larger number of events) risk. For example, age <40 that is protective against both risks may yield higher cumulative incidences regarding the minor risk than is observed in those age 40 - 60. The potential for misleading information makes it undesirable to interpret a covariate effect on a minor risk. In our study, there were 200 ESRD patients and 1482 deaths without ESRD, and the two risks are highly unbalanced. This is a good example of how use of the subdistribution hazards model on ESRD for a minor risk is not reliable, and why people should be cautious in interpreting such analysis. Thus, direct assessment of the covariate effect in the subdistribution model should not be conducted on the cumulative incidence of the minor risk. For death without ESRD as the dominant risk, one can use either a cause-specific hazard or subdistribution hazard model. However, for a minor risk, only the Cox cause-specific hazards model appears reliable.

The cause-specific hazard can be modeled using the Cox model, which is broadly used in medical research. The cause-specific hazard model may be more clinically understandable when assessing the prognostic effect of the covariates on a specific cause because we see that the covariate effect would be to reduce or increase the instantaneous probability of the event of interest irrespective of other covariate effect. However, when the study objective is to compare the probability of the event of interest, then the subdistribution hazards model will be appropriate. While the subdistribution hazards model might be limited to populations with similar characteristics and similar competing risk rate, the cause-specific hazard model is applicable for any population with similar characteristics regardless of the rates of competing risk events [39]. A cause-specific hazard can be expressed graphically, but they are not easy to interpret. Additional issues arise with interpretation of covariates on the hazard scale. The Cox subdistribution hazards model provides a methodology for modeling CIF with covariates using a proportional hazards assumption. The CIF are well suited to summarize competing risks data with a graphic display of the probabilities of event causes against time [8]. The CIF curve derived from a cause-specific hazard function provides the probability of failure due to the event of interest in competing risks analysis [16,37-40].

An advantage of the Lunn and McNeil approach is that it facilitates direct comparisons between different event types. Depending on whether the assumption of proportional baseline cause-specific hazards holds, an unstratified or a stratified Cox regression could be applied. The unstratified method assumes that the baseline hazards for different risk types are proportional, while the stratified one allows for different hazards in each event type [41]. When the assumption of proportional baseline cause-specific hazards is satisfied, interpretation of the estimates from the unstratified Lunn and McNeil model is straightforward and allows assessment of the relative clinical importance of different event types. An advantage of the L-M model compared to the Cox cause-specific model is the flexibility to perform statistical inferences about various features of the competing risks using the information directly provided in the computer output. However, for the unstratified L-M model the constant assumption must be held within strata, otherwise the model is not valid. In most studies, different competing risk types will have substantially different underlying hazard functions and thus the applicability of the unstratified L-M model is restricted. Another limitation is that carrying out the L-M model requires additional data layout coding [42].

The cause-specific and subdistribution models share the same proportional hazards assumptions but normally the covariate effects on the cause-specific hazards and on the subdistribution hazards models are different [37]. This occurs because the effect of a covariate on the cumulative incidence of a particular cause is mediated via its direct effect on the cause specific hazard of that cause and via its indirect effect on the cause specific hazards for other cause [43]. Regarding the covariate effects, the results of the subdistribution hazards model have similar interpretations compared to the Cox model approach for competing risk data analysis: *e^β ^*represents the increase of the hazard of the subdistribution due to one unit increase of *z*. However, the cause-specific models

do not allow for a probability interpretation because the cumulative probability depends on other cause-specific events. Thus, a summarizing the probability of the different effects of the cause-specific hazards is challenging [2,43,44]. Nonparametric inference for general summary measures for differences on the cumulative incidence functions was proposed [43]. It had also been shown that a proportional subdistribution hazards model provides an interpretable summary when the overall effects of covariate on the CIF are of interest [2]. Under the non-proportional subdistribution hazards, the estimated subdistribution hazards ratio for the CIFs is also interpretable as a time-averaged hazard ratio [44,45]. Further, even if the proportional subdistribution hazards model is misspecified, it provides an interpretable summary of separate cause-specific analyses [44].

The cause-specific and subdistribution models both require assumptions of proportional hazards. The proportionality assumption can be checked by evaluating the log{- log *S*_*KM*_*(t)*} or by plotting residuals (Cox-Snell , Martingale, or Deviance residuals) or by adding time-dependent covariates in the model [12,35,46]. For the Cox cause-specific hazard model, the statistical software is available in many commercial statistical software packages and makes it easy to fit the models. However, for the sub distribution hazard models, currently standard procedure is not available in SAS, but SAS macros [47], STATA with ***compet.ado ***or **R**-package ***cmprsk ***are available.

In our study, we used data from administrative databases to estimate the competing risks of ESRD and mortality in First Nations people with diabetes. Since it was not a prospective study design and the subjects' clinical characteristics were not available, other important risk factors for ESRD and mortality could not be assessed. If the study had access to patients' demographics beyond age, gender and ethnicity and clinical information such information could be incorporated in the competing events analyses of ESRD and death. Interplay between the competing risks of ESRD and death might give the complete story about the effects of risk factors. If risk factors are different for two competing events then it is necessary to examine the decomposed outcomes on ESRD and mortality since pathways to the two events may be different. Establishing risk factors that cause progression to ESRD and distinguishing such risk factors from those that increase mortality can clearly predict two endpoints of ESRD and death, and can also be used as a decision making instrument.

## 6. Conclusion

In the analysis of competing risk data it is important to present both the results of the event of interest and the results of competing risks. One can use either the cause-specific hazards model or the subdistribution hazards model for a dominant risk. However, for a minor risk we do not recommend the subdistribution hazards model and a cause-specific hazards model is more appropriate in competing risk data analysis. In interpreting the results of a competing risks analysis, we should always take into account all causes. Focusing the interpretation on one or a few causes and ignoring the other causes is always associated with a risk of overlooking important features which may influence our interpretation. Investigators should take care in setting up the right models to answer the questions of interest in their research. A graphic illustration of CIF curves will provide important additional insight, although the statistical tests like the log-rank test remains appropriate for testing group differences on each event type. Applying them together as complementary measures of risk clearly can expand a decision-making instrument for many competing risks studies.

## Competing interests

The authors declare that they have no competing interests.

## Authors' contributions

HJL conducted the literature review, developed the mathematical framework, derived the results, and prepared the manuscript. XZ wrote computer programs and produced graphs.

RD defined the original study populations, acquired the data and provided advice on how to relate the topic to previous work in the field. NO reviewed the work and gave important editorial suggestions that greatly improved the manuscript. All authors have read and approved the final manuscript.

## Pre-publication history

The pre-publication history for this paper can be accessed here:

http://www.biomedcentral.com/1471-2288/10/97/prepub
